# Database quality assessment in research in paramedicine: a scoping review

**DOI:** 10.1186/s13049-023-01145-2

**Published:** 2023-11-11

**Authors:** Neil McDonald, Nicola Little, Dean Kriellaars, Malcolm B. Doupe, Gordon Giesbrecht, Rob T. Pryce

**Affiliations:** 1 Winnipeg Fire Paramedic Service, EMS Training, 2546 McPhillips St, Winnipeg, MB R2P 2T2 Canada; 2 Department of Emergency Medicine, Max Rady College of Medicine, University of Manitoba, S203 Medical Services Building, 750 Bannatyne Ave, Winnipeg, MB R3E 0W2 Canada; 3 Applied Health Sciences, University of Manitoba, 202 Active Living Centre, Winnipeg, MB R3T 2N2 Canada; 4 College of Rehabilitation Sciences, Rady Faculty of Health Sciences, University of Manitoba, 771 McDermot Ave, Winnipeg, MB R3E 0T6 Canada; 5 Department of Community Health Sciences, Rady Faculty of Health Sciences, University of Manitoba, 750 Bannatyne Ave, Winnipeg, MB R3E 0W2 Canada; 6 Faculty of Kinesiology and Recreation Management, University of Manitoba, 102-420 University Crescent, Winnipeg, MB R3T 2N2 Canada; 7 Department of Kinesiology and Applied Health, Gupta Faculty of Kinesiology, University of Winnipeg, 400 Spence St, Winnipeg, MB R3B 2E9 Canada

**Keywords:** Paramedicine, Prehospital, Data, Data quality, Emergency medical services, Data collection, Medical records, Electronic health records

## Abstract

**Background:**

Research in paramedicine faces challenges in developing research capacity, including access to high-quality data. A variety of unique factors in the paramedic work environment influence data quality. In other fields of healthcare, data quality assessment (DQA) frameworks provide common methods of quality assessment as well as standards of transparent reporting. No similar DQA frameworks exist for paramedicine, and practices related to DQA are sporadically reported. This scoping review aims to describe the range, extent, and nature of DQA practices within research in paramedicine.

**Methods:**

This review followed a registered and published protocol. In consultation with a professional librarian, a search strategy was developed and applied to MEDLINE (National Library of Medicine), EMBASE (Elsevier), Scopus (Elsevier), and CINAHL (EBSCO) to identify studies published from 2011 through 2021 that assess paramedic data quality as a stated goal. Studies that reported quantitative results of DQA using data that relate primarily to the paramedic practice environment were included. Protocols, commentaries, and similar study types were excluded. Title/abstract screening was conducted by two reviewers; full-text screening was conducted by two, with a third participating to resolve disagreements. Data were extracted using a piloted data-charting form.

**Results:**

Searching yielded 10,105 unique articles. After title and abstract screening, 199 remained for full-text review; 97 were included in the analysis. Included studies varied widely in many characteristics. Majorities were conducted in the United States (51%), assessed data containing between 100 and 9,999 records (61%), or assessed one of three topic areas: data, trauma, or out-of-hospital cardiac arrest (61%). All data-quality domains assessed could be grouped under 5 summary domains: completeness, linkage, accuracy, reliability, and representativeness.

**Conclusions:**

There are few common standards in terms of variables, domains, methods, or quality thresholds for DQA in paramedic research. Terminology used to describe quality domains varied among included studies and frequently overlapped. The included studies showed no evidence of assessing some domains and emerging topics seen in other areas of healthcare. Research in paramedicine would benefit from a standardized framework for DQA that allows for local variation while establishing common methods, terminology, and reporting standards.

**Supplementary Information:**

The online version contains supplementary material available at 10.1186/s13049-023-01145-2.

## Background

Paramedicine^1^ is increasingly recognized as a distinct healthcare profession with a unique body of knowledge [1–3]. Numerous studies and position papers have cited the need for research to develop quality benchmarks, investigate interventions, and evaluate outcomes within paramedic practice [2, 4–8]. While research in paramedicine continues to grow and evolve, the field faces key barriers to its ongoing development [6, 9, 10]. Among these, access to high-quality records of paramedic clinical practice (hereafter, paramedic data) has been identified as a critical barrier to linking to patient outcomes and researching paramedic care [2, 7, 8, 11].

The paramedic practice environment poses unique challenges to data collection [5, 10, 12–14]. Paramedic work is dynamic and complex, and takes place in uncontrolled and unpredictable environments, often subject to time and other pressures. Data collection is frequently delayed or shared among practitioners also providing care, resulting in potential data loss or inaccuracy [14, 15]. Records of paramedic care, historically paper-based, are transitioning to electronic platforms, but face continuing challenges to implementation in many jurisdictions [16, 17]. Paramedic services (as well as other emergency response agencies) typically organize documentation based on the incident, not the patient. Incident-based record keeping then requires linkage to subsequent files to assess outcomes for individual patients [18]. Data linkage using paramedic records varies in terms of success, not least in relation to the quality of initial data, and the linkage process can be susceptible to various forms of bias [11, 19].

Electronic health records in all contexts have benefits and limitations, but all require consistent ways of describing, assessing, and integrating information about data quality [20–22] . These needs apply equally to paramedic data. Despite challenges to data collection and analysis, research capacity in paramedicine will depend on consistent and valid methods of data collection, as well as a common language of quality assessment and standards of transparent reporting. Other healthcare professions have addressed these goals by developing conceptual tools for assessing data quality [23, 24]. Usually termed data quality assessment (DQA) frameworks, these tools provide both templates for data evaluation and guidance for future data collection. They establish baseline methodological standards, which in turn support the methodological quality of future research and the validity of results.

DQA frameworks cover a wide range of settings and purposes. Typically, they are organized by domains – distinct aspects of data that together make up a total picture of data quality in any particular field. The number of domains included in any framework can vary widely, and the terms used to describe similar concepts frequently overlap. Although as many as 49 different domains have been described in one practice area, [25]. frameworks typically include between one and eight domains, with key concepts such as completeness, accuracy, and timeliness appearing most frequently [25]. These and similar examples of domains from other healthcare disciplines have not been adopted in paramedicine. Although some position statements on data capture and reporting have been published, [26, 27]. no comprehensive framework dedicated to the paramedic work environment has been developed, and the adaptability of existing ones to the unique circumstances of paramedicine has not been determined.

As paramedic research continues to evolve, studies that rely on records of paramedic clinical practice will require a common language and standard of data assessment to support methodological rigor. In the absence of a paramedic-specific DQA framework, the landscape of data-quality practices remains uncharted. No prior reviews have collected information on this topic, and reporting of DQA practices within paramedicine remains sporadic. Currently, we lack a comprehensive view of what data are assessed, methods for doing so, and the ensuing results. Recognizing a need to understand the extent to which paramedicine researchers have embedded information about data quality into their research products, this manuscript describes the results of a scoping review that was conducted to describe the range, extent, and nature of DQA practices reported in paramedicine research.

## Methods

A protocol of the methods has been previously registered with the Open Science Framework (https://doi.org/10.17605/OSF.IO/Z287T) and published [28]. Reporting follows the guidelines of the Preferred Reporting Items in Systematic Reviews and Meta-Analyses extension for scoping reviews (PRISMA-ScR) [29].

### Aim

This scoping review asks, what are the range, extent and nature of DQA practices in paramedic research? It aims to document these characteristics to support ongoing development of methodological standards in research in paramedicine.

### Search strategy

With the support of a professional librarian and in accordance with established methods, a search was constructed to reflect the population, context, and concept of the research question [30, 31]. Paramedic research studies that assessed data quality as a major goal and reported quantitative DQA results from the paramedic practice environment were included. This environment included urban, rural, remote, and military settings, but excluded special circumstances (disaster and mass-casualty situations). Studies were excluded if they were protocols, commentaries, case studies, interviews, simulations, or used experimental data-processing techniques. Studies that were not primarily concerned with paramedic data, or studies that evaluated databases that incidentally included paramedic information, were also excluded. No restrictions were placed on language. After iterative refinement of search terms and pilot testing of date ranges, the search was limited to 2011–2021 to balance comprehensiveness with recency. The search was applied to the following databases: MEDLINE (National Library of Medicine), Embase (Elsevier), Scopus (Elsevier) and CINAHL (EBSCO). The searches as applied are available in “Additional file 1” and reflect the specific terminology, logical combinations, and formatting of each database. Generic keywords and subject headings are listed for illustration: “emergency medical services”, “emergency medical technicians”, “ambulance”, “paramedic”, “paramed*”, “prehospital”, “first respond*”, “emergency services”, “quality improvement”, “quality assurance”, “health care”, “information storage”, “information retrieval”, “data collection”, “medical records”, “electronic health records”, “health records, personal”, “medical record linkage”, “medical records systems, computerized”, “patient regist*”, “data quality”, “electronic medical record”, “record linkage”, “paramedic record”.

### Screening

Search results were imported into a data-management program (Covidence, Veritas Health Innovation, Melbourne, Australia). After duplicate citations were removed, all authors participated in title and abstract screening of 250 records to practice and discuss the application of inclusion criteria. All remaining records were independently screened by at least two reviewers, and any record selected by any reviewer was retained for full-text screening. Full-text records were assessed independently by two reviewers (NM, RP); differences were resolved with discussion, including the third reviewer (NL).

### Data extraction

Data were extracted using a custom-designed data-extraction form (“Additional file 2”). This form included 13 fields grouped according to the range, extent, and nature of DQA practices. Range was defined by geographic location, year of publication, study purpose, and topic (whether a clinical area, population, or specific circumstance). Extent was documented by the level, breadth, and number of records assessed. Within extent, level refers to the organizational area of the primary data and includes five categories: local (municipal or small area); regional (such as a regional health authority); sub-national jurisdiction (state/province/county); national; and international. Breadth contains two components: the number of services included and the number of linkages between paramedic data and other types of databases. The nature of the DQA was summarized by the specific variables or fields assessed, the methods of assessment, results, the domain of data quality being assessed, and the presence of any quality threshold. In accordance with guidance on scoping reviews, critical appraisal was not performed [29]. Data were extracted iteratively, and key information was summarized for reporting, either quantitatively or with representative examples.

### Protocol amendments

These methods correspond to the registered study protocol with following exceptions. Each change was based on the consensus of reviewers during data extraction. (1) The duration of data assessed was replaced by number of records assessed. (2) A field to record any quality threshold or summary rating of data quality was added. (3) The study protocol called for data-quality domains to be recorded both as identified by the study, and according to an existing framework used by the Canadian Institute for Health Information (CIHI) [32]. Since the included studies used a wide variety of descriptions to identify assessment domains, terms covering similar concepts were grouped under the domain name that was most applicable or appeared most frequently (with all alternative terms listed). As well, categorization of assessment domains under the CIHI framework yielded only two categories (Accuracy & Reliability and Comparability & Coherence). As these results added little interpretive value, they have not been reported.

## Results

**Fig. 1:**
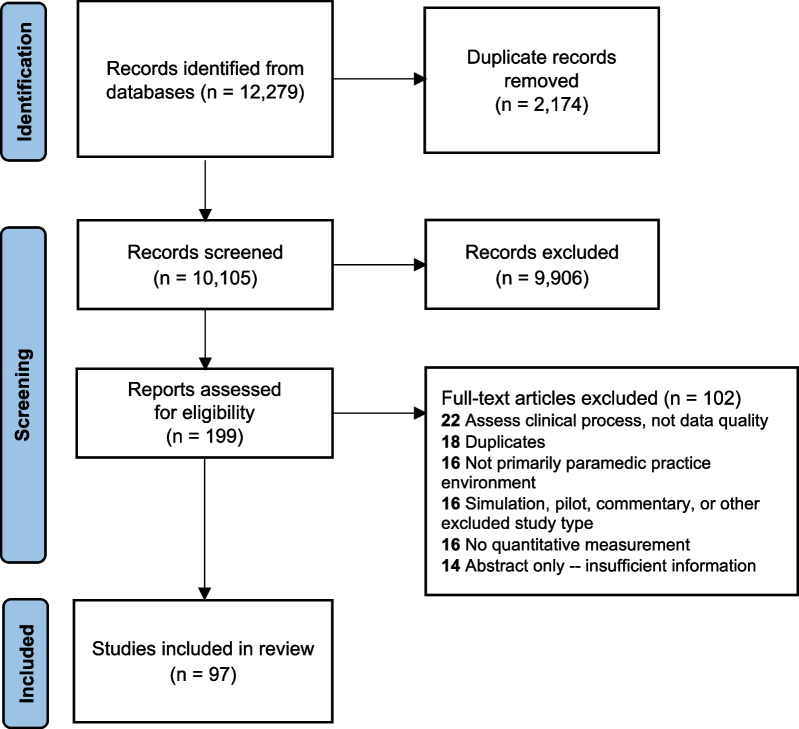
PRISMA-ScR flow diagram of study selection

Database searching identified 10,105 unique articles (Fig. 1). After title and abstract screening, 199 remained for full-text review. Of these, four were in languages other than English (one each of German, Spanish, Russian, and Persian [Farsi]); these were professionally translated for further assessment. Among all articles selected for full-text assessment, 102 were excluded for reasons cited. Additional duplicates (*n* = 18) identified at this stage included abstracts for which full articles using the same data and substantially similar results were also present. Ninety-seven articles were included in the analysis.

### Study characteristics

Table 1 lists the main characteristics of included studies, as well as selected extracted data. (“Additional file 3” lists full citations of all included studies.)

**Table 1:** Characteristics of included studies [inserted at end of document]

	Range			Extent				Nature
Study	Year	Location	Topic	Level of data**	Number of services	Number of linkages	Number of records (10^*x*)***	Domains assessed (as summarized)
Abir et al	2021	USA	Data	Sub-national	10 or more	None	6	Completeness
Alstrup et al	2019	Denmark	Data	National	1	None	4	Completeness, Accuracy
Andrews et al	2019	Australia	Trauma (MVCs)	Sub-national	2 to 9	None	3	Completeness, Reliability
Andrusiek et al.*	2012	Canada	Police use of force	Local	1	None	2	Linkage
Asimos et al	2014	USA	Stroke	Sub-national	10 or more	None	3	Completeness
Babcock et al.*	2019	USA	Sepsis, Pediatrics	Regional	10 or more	One	3	Completeness
Barley et al.*	2021	UK	Vitals / History	Regional	1	None	5	Completeness
Berben et al	2015	Holland	Pain	Regional	10 or more	None	3	Completeness
Bergrath et al	2011	Germany	Data	Local	1	None	3	Completeness
Bessant et al.*	2017	UK	Trauma (tourniquets)	National	2 to 9	None	1	Completeness
Betlehem et al.*	2013	Hungary	Stroke	Sub-national	1	One	2	Completeness
Blanchard et al	2021	Canada	Data	Regional	1	One	3	Linkage, Representativeness
Bloomer et al	2013	Australia	Airway (intubation)	Local	1	None	2	Completeness
Bradley et al	2017	Canada	Trauma	Sub-national	1	None	2	Completeness
Carroll et al	2015	Australia	Data	Sub-national	1	Multiple	6	Linkage
Chikani et al	2020	USA	Data	Sub-national	10 or more	One	5	Linkage
Clark et al	2019	UK	Data	Regional	1	One	5	Linkage
Coventry et al	2014	Australia	Other cardiac	Local	1	One	2	Completeness, Accuracy, Reliability
Cox et al	2013	Australia	Data	Sub-national	1	None	3	Linkage
Crilly et al	2011	Australia	Data	Sub-national	1	One	4	Linkage
Cunningham et al	2014	Australia	Trauma (falls)	Regional	1	One	2	Completeness
Deasy et al	2013	Australia	OHCA, Pediatrics	Sub-national	1	One	2	Linkage
Demel et al.*	2018	USA	Stroke	Sub-national	10 or more	One	4	Completeness
Depinet et al	2019	USA	Sepsis, Pediatrics	Regional	10 or more	One	2	Completeness, Reliability
Dewolf et al	2021	Belgium	OHCA	Local	1	One	2	Accuracy
Engels et al	2021	Canada	Trauma	Regional	2 to 9	Multiple	4	Linkage
Fein et al	2014	Australia	Trauma (burns), Pediatrics	Regional	1	None	2	Completeness
Fix et al	2021	USA	Substance use	Sub-national	10 or more	One	4	Linkage
Fosbol et al	2013	USA	Other cardiac	Sub-national	10 or more	One	3	Linkage, Reliability, Completeness
Foster et al	2017	USA	OHCA	Local	1	One	3	Completeness, Accuracy
Frisch et al	2014	USA	OCHA	Local	2 to 9	None	2	Accuracy, Reliability
Gaeeni et al	2021	Iran	Data	Sub-national	1	None	2	Completeness
GarciaMinguito et al	2012	Spain	Trauma (domestic violence)	Local	2 to 9	None	2	Completeness
Gerhardt et al	2016	USA	Pain	Regional	1	One	3	Completeness
Govindarajan et al.*	2011	USA	Data	Regional	2 to 9	Multiple	3	Linkage
Gravens et al.*	2018	USA	OHCA, Vitals / History	Local	1	One	2	Completeness
Halbesma et al.*	2019	UK	OHCA	National	1	Multiple	4	Linkage
Hern et al.*	2012	USA	Pain	Local	1	None	4	Completeness
Hu et al	2014	USA	Trauma, Vitals / History	Sub-national	1	None	2	Reliability, Completeness
Hughes-Gooding et al	2020	UK	Seizures	Regional	1	Multiple	5	Linkage
Ibrahim et al.*	2019	USA	Stroke	Sub-national	10 or more	One	3	Linkage
Jaureguibeitia et al	2021	USA	OHCA	National	10 or more	None	3	Representativeness, Accuracy
Ji et al	2018	UK	OHCA	Regional	2 to 9	Multiple	3	Linkage
Katzer et al	2012	USA	Data	Local	1	None	2	Completeness
Kearney et al	2016	Rwanda	Trauma	Local	1	One	3	Linkage
Ko et al.*	2012	Unknown	OHCA	Local	1	One	3	Completeness
Kummer et al	2017	USA	Stroke	Local	1	None	1	Completeness
Lerner et al	2014	USA	Pediatrics	National	10 or more	None	5	Completeness
Lerner et al	2021	USA	Pediatrics	National	10 or more	None	5	Representativeness
Li et al.*	2016	Unknown	Vitals / History, Geriatrics	Local	1	One	2	Completeness
Lippert et al.*	2019	Denmark	OHCA	National	2 to 9	One	3	Completeness
MacDougall et al	2019	Canada	Substance use	Sub-national	1	Multiple	3	Linkage
Mann et al	2015	USA	Data	National	10 or more	None	3	Completeness, Representativeness
McDonald et al.*	2020	USA	OHCA	Local	1	One	1	Linkage
Miller et al	2021	USA	Data	National	10 or more	None	6	Representativeness
Mumma et al	2015	USA	OHCA	Sub-national	10 or more	Multiple	4	Linkage
Mysliwiec et al.*	2015	USA	Geriatrics	Local	1	None	2	Completeness
Newgard et al	2011	USA	Data	Sub-national	10 or more	One	4	Completeness, Linkage
Newgard et al	2012	USA	Data, Trauma	Regional	10 or more	Multiple	4	Completeness, Linkage
Newgard et al	2012	USA	Data, Trauma	Regional	10 or more	Multiple	4	Accuracy, Reliability
Newgard et al	2018	USA	Data, Trauma, Geriatrics	Regional	10 or more	Multiple	4	Completeness, Accuracy, Linkage
Nishiyama et al	2014	Unknown	OHCA	International	10 or more	None	5	Completeness
Oostema et al	2020	USA	Stroke	Sub-national	10 or more	One	3	Linkage, Representativeness
Oud et al	2019	Australia	Airway (intubation)	Local	1	None	1	Completeness
Outterson et al.*	2016	Unknown	Other cardiac, Vitals/History	Local	1	One	2	Reliability
Perez et al.*	2017	USA	Trauma (TBI)	Regional	2 to 9	One	2	Accuracy
Perez et al.*	2017	USA	Trauma (TBI)	Regional	2 to 9	One	2	Accuracy
Poulsen et al	2020	Denmark	Vitals / History	Regional	1	None	5	Completeness, Accuracy
Rajagopal et al	2017	UK	OHCA	National	2 to 9	Multiple	4	Linkage
Randell et al.*	2020	USA	Data	Local	1	None	2	Completeness
Redfield et al	2020	USA	Data	Local	1	One	4	Linkage
Reisner et al	2012	Unknown	Trauma, Vitals / History	Local	1	One	2	Reliability
Richards et al.*	2018	USA	Stroke	Local	1	One	2	Linkage
Robinson et al	2016	USA	Trauma	Regional	1	One	2	Completeness
Rykulski et al.*	2021	USA	OHCA	Sub-national	10 or more	One	3	Completeness
Savary et al.*	2020	France	OHCA	Regional	2 to 9	One	2	Completeness
Saviluoto et al	2020	Finland	Data	International	1	None	5	Completeness
Schauer et al	2017	USA	Trauma—all	Regional	1	One	2	Completeness
Scott et al	2013	USA	Trauma	Sub-national	10 or more	One	2	Linkage
Seymour et al	2014	USA	Data	Regional	10 or more	Multiple	3	Linkage, Representativeness Completeness
Silvestri et al.*	2012	USA	Airway (intubation)	Local	1	None	2	Accuracy
Staff et al	2011	Norway	Trauma (MVCs)	Sub-national	10 or more	None	2	Completeness, Reliability, Representativeness
Stephanian et al.*	2020	Canada	Mental Health, Trauma (falls)	Local	1	Multiple	3	Linkage, Representativeness
Stromsoe et al	2013	Sweden	OHCA	Sub-national	2 to 9	None	3	Completeness, Representativeness
Sundermann et al	2015	USA	OHCA	Local	1	None	3	Completeness, Accuracy
Swor et al	2018	USA	OHCA	Sub-national	10 or more	One	4	Linkage
Tonsager et al	2019	Multinational	Data	International	2 to 9	None	3	Completeness
Tonsager et al	2020	Multinational	Vitals / History	International	10 or more	None	4	Accuracy, Completeness, Representativeness
Tainter et al	2020	USA	Trauma (MVCs)	Sub-national	10 or more	One	4	Linkage
Therien et al	2011	USA	Trauma (combat)	Regional	1	One	4	Completeness
Timoteo et al	2020	Brazil	Data	Local	1	None	2	Completeness
Tlimat et al.*	2016	USA	Data	Local	1	One	4	Linkage
Tsur et al	2020	Israel	Trauma (combat)	National	1	One	4	Completeness
Wilharm et al	2019	Germany	Airway (capnometry)	International	10 or more	One	4	Completeness
Winter et al.*	2017	UK	Pain	Regional	1	None	2	Completeness

*Denotes abstract

**Sub-national refers to state/province/county, as per article

***The number of records is expressed as an order of magnitude. For example, "3" represents 10^3, meaning between 1,000 and 9,999 records

ACS Acute coronary syndrome, MVC Motor vehicle crash, MI Myocardial infarction, N/A Not applicable, OHCA Out-of-hospital cardiac arrest, STEMI ST-elevation myocardial infarction, TBI Traumatic brain injury, UK United Kingdom, USA United States of America

### Range of included articles

**Fig. 2:**
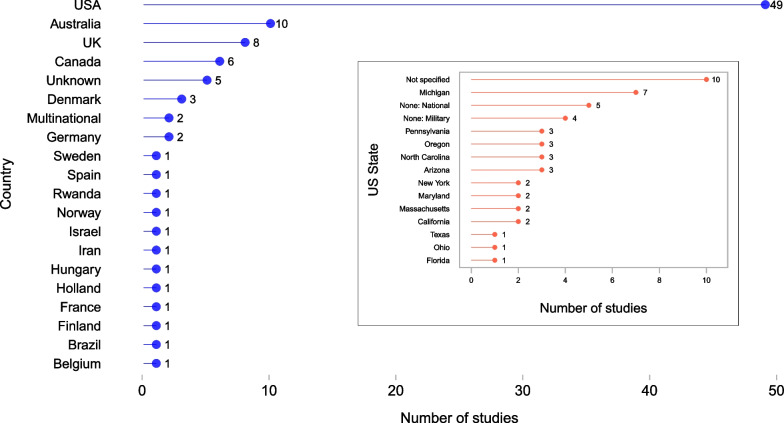
Geographic location of data quality assessment studies in research in paramedicine (*n* = 97), listing the number of studies by country (main panel), and by State (or national / military) among studies from the United States (inset)

**Fig. 3:**
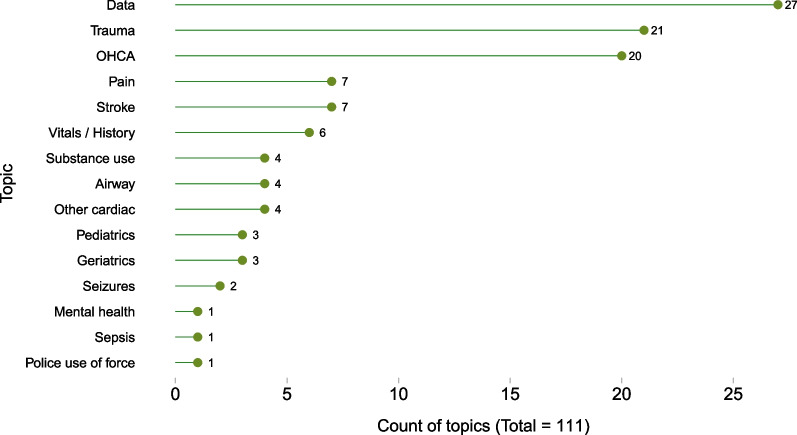
Topic (clinical area / population / situation) of data quality assessment studies in research in paramedicine, listing the number of areas (total = 111) among all studies (*n* = 97)

Among the 97 included articles, 39 (40%) were published from 2019 to 2021, with the remainder spread relatively evening across the preceding years. Forty-nine studies (51%) were conducted in the United States (US); Australia (*n* = 10), the United Kingdom (*n* = 8), and Canada (*n* = 6) were the next most frequent locations. Figure 2 lists all countries, as well as the breakdown of US States, where applicable. Abstracts (as well as one letter) accounted for 27 (28%) included items; the remainder (*n* = 70, 72%) were full articles. Included articles studied diverse topics spanning clinical areas, populations, and specific situations. Studies were coded to allow for multiple subject areas; Fig. 3 illustrates the number of studies per topic out of all mentioned (*n* = 111). Topics related to data linkage or the data management without reference to a clinical area (labelled, “Data”) were the most frequent area of study (*n* = 27, 24%). The next most common topic was trauma (*n* = 21, 19%), followed by out-of-hospital cardiac arrest (OHCA) (*n* = 20, 18%). These three areas made up the majority (68/111, 61%) of all areas studied.

### Extent of included studies

**Fig. 4:**
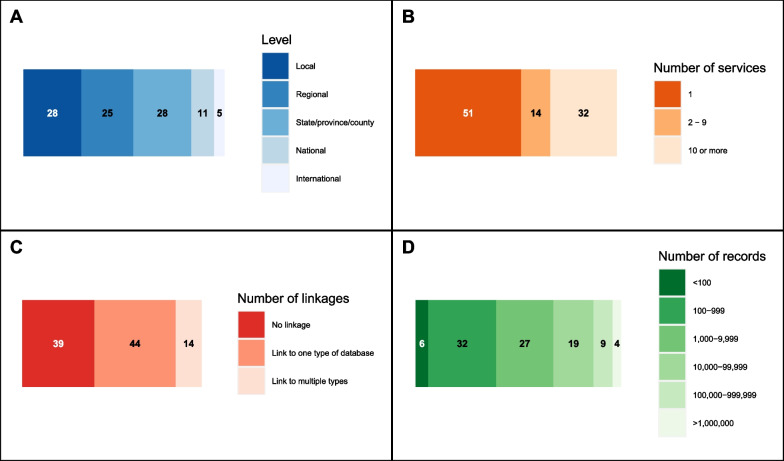
The extent of data quality assessment studies in research in paramedicine, measured by **A** the level of data assessed, **B** the number of services included, **C** the number of types of linkages to other databases, and **D**, the number of records assessed. Each chart includes all studies (*n* = 97)

Figure 4 displays the extent of included studies according to the identified sub-categories. The level at which studies assessed data was spread relatively evenly among local (*n* = 28, 29%), regional (*n* = 25, 26%), and state/province/county (*n* = 28, 29%) (Fig. 4A). The majority of studies (*n* = 51, 53%) assessed data belonging to one paramedic or prehospital agency (Fig. 4B). In terms of linkage, 39 (40%) studies did not link paramedic or prehospital data to any other sources, whereas forty-four (45%) linked to a single type of database (whether hospital, emergency department, or other related source), and 14 (14%) linked to multiple databases of different kinds (Fig. 4C). The majority of studies reviewed between 100 and 9,999 records (*n* = 59, 61%), with only 6 (6%) reviewing fewer than 100 and 4 (4%) reviewing more than 1 million (Fig. 4D). Considering combinations of the level of data assessed (Fig. 4BA), the number of services (Fig. 4B), and number of linkages (Fig. 4C), the three largest exclusive groups of characteristics involved: a local, single service linked to a single type of database (13/97); state-level data, represented by 10 or more services, linked to a single type of database (13/97); and a local, single service with no linkage (12/97).

### Nature of included studies

Table 2 summarizes the domain names and explanations derived from how the studies described their assessment. It also includes any quality measures applied by included studies, grouped by domain. As listed in Table 1, some studies assessed multiple areas, yielding 126 instances of an assessed domain.

**Table 2:** Summary of data quality assessment domains in studies on research in paramedicine

Domain	Count (percent)*	Other terms used	Description	How measured	Quality measure
Completeness	57 (45)	Missingness, adherence, availability, unknown/not reported, granularity	Measure of how often a variable is present when expected. Complement of missingness	Proportion and percent	Raw percent complete, weighted percent complete, percent legible
Linkage	34 (27)	Match	Can records belonging to the same person or event be linked between different databases? How well? By what means?	Probabilities, percent success, sensitivities, specificity, positive predictive value, negative predictive value (and related measures: false positive, false negative)	Match-weight cut-off, match quality
Accuracy	14 (11)	Validity, correctness, concordance, plausibility, ascertainment, capture, incidence, population	Does the variable measure what it claims to measure? Is the result plausible or possible?	Proportion and percent, sensitivity, specificity, positive predictive value, negative predictive value	–
Reliability	10 (8)	Agreement, precision, consistency, variation, aggregation, uniqueness, granularity, quality	Is the measurement free from error and consistent over time and among observers?	Difference in proportations, kappa, intraclass correlation coefficient, correlation, other (Andrews, Reisner)	–
Representativeness	11 (9)	External validity, bias, generalizability, concordance	How well does the data correspond to other data expected to be similar? How well do parts of the data correspond when they are expected to be similar? Is the data biased in some way?	Difference in proportions, correlation, kappa, sensitivity	Proportions, absolute standardized difference, ± 5% difference

*126 domains assessed among 97 studies

As incidental findings, one study adapted a DQA framework from public-health surveillance and applied some domains to its prehospital data [33]. Similarly, two studies applied a reporting guideline specific to the methodology of database linkage [34, 35]. No other DQA reporting guidelines were noted.

The DQA domains of the included studies are summarized below, with examples of representative and unique studies.

### Completeness

The included studies used a variety of terms that can be summarized as assessments of completeness (Table 2). Based on the practices described, completeness measured how often a variable was present when expected or required. It was usually expressed as a proportion or percent of all potential entries. Depending on the purpose of the study or the nature of the results, this was often represented as its complement, missingness. This domain appeared most frequently, and was present in 57 studies, accounting for 45% of all domains documented (*n* = 126).

Among included studies, completeness frequently measured the variables deemed most important to each study’s purpose. For example, Abir et. al. found only five of 18 key variables were present in over 90% of cases [36]. Other large studies provided similar ranges, [37] although some report wide discrepancies among individual services in aggregated data [38, 39] . Certain categories, such as mechanism of injury, frequently showed relatively low values [40]; emergency department (ED) disposition, where reported, was negligibly complete in paramedic databases (cited in one study at less than 5% [41]). Additional contrasts in the completeness of basic variables can be seen between different settings, such as helicopter emergency medical services (EMS) agencies and the military, where completion rates were consistently high and low, respectively [42–45].

### Linkage

Thirty-four studies (representing 27% of all domains) assessed how well paramedic or prehospital data could be linked to other sources of information. Included studies detailed a range of techniques for linkage, broadly divided between deterministic and probabilistic approaches, occasionally supplemented by manual review for confirmation or optimization [46, 47].

Overall rates of linkage varied among the included studies. In one case, an optimized iterative deterministic approach yielded 97% success in linking records of EMS patients transported to an ED, with no false positives [19]. Other studies found similar results with a variety of optimization strategies [35, 48–50]. Contrasting results appeared in several studies linking trauma patients to hospital outcomes, ranging between 15 and 88%, and 49–60% specifically for ground transport [51, 52]. Others examining OHCA (34% [53]) and stroke (26% [54]) marked the lowest reported rates within those clinical areas.

### Accuracy

Among a range of terms used by the included studies to describe similar concepts, accuracy summarizes practices that evaluated the extent to which a variable recorded what it was designed to measure. When it was assessed, accuracy was measured against a reference thought to be valid or true, sometimes referred to as a gold standard. It was expressed in terms of proportions, percents, and diagnostic test statistics (sensitivity, specificity, positive predictive value, negative predictive value). Evaluations of accuracy were present in 14 studies, accounting for 11% of all domains assessed.

Several topics featured multiple studies assessing accuracy, including OHCA, [55–57] vital-sign documentation, [58, 59] [37, 60] and patient history. [61, 62] Within OHCA, three studies evaluated the accuracy of documented events and timepoints in the paramedic record in comparison to video or audio recordings or data from a defibrillator/monitor – in each case, a source thought to represent a gold standard. All showed discrepancies between written and recorded data, including, for example, detection of return of spontaneous circulation and re-arrest, [56] the rate and depth of chest compressions, [57] and total CPR time and total adrenaline dose [55].

### Reliability

In addition to assessing accuracy, some included studies also measured the extent to which measurements and documentation were consistent or how much variety would appear over repeated measures. This was most commonly described as reliability, although agreement, consistency, and other terms were used for the same domain (Table 2). In contrast to measures of accuracy and validity, reliability assessed agreement between two values without assuming that one represented a reference standard. In place of statistics that measure proximity to a value, reliability was expressed in terms of correlation, kappa, intraclass correlation coefficient, difference, differences in proportions, and unique measures derived by individual studies [40, 63]. Ten studies presented quantitative data falling under these headings, representing 8% of domains evaluated.

Whereas several studies evaluated the accuracy of prehospital documentation of patient medical history in comparison to hospital records, some analyses assessed the same information in terms of agreement. For example, Coventry et. al. found that paramedic and hospital documentation showed high agreement in recording the presence of chest pain among patients with myocardial infarctions (adjusted kappa, *k* = 0.87).[62].

When applied specifically to the spread or clustering of measurements, reliability is commonly termed precision. (This was also referred to as granularity in the case of time stamps [40].) In assessing documented event times in OCHA in comparison to audio recordings, Frisch et al*.* found wide variability in reported times – imprecision that they argue should be accounted for in future analyses [64]. Precision has also been assessed in terms of how many different ways variables are recorded, both within and across datasets. Staff et al*.* examined whether vital signs in trauma calls were recorded as exact numbers, categories, or inferred from free-text [65] . Common variables recorded differently both within and across datasets were cited in other instances, including vital signs, [66] chief-complaint coding among different services, [38], and even ostensibly standardized variables in OHCA reporting [67].

### Representativeness

Studies that examined the extent to which data corresponded to reference populations or to the degree to which data could be applied outside of the study group assessed representativeness (or generalizability, bias, concordance, or external validity). Among included articles, representativeness was assessed most often by comparisons of proportions, although correlation, agreement, and unique statistics were also used [68] Eleven studies included assessments of representativeness, accounting for 9% of domains.

Studies in paramedic research used a variety of approaches to defining a reference group. Mann et. al. assessed the generalizability of the 2012 National Emergency Medical Services Information System (NEMSIS, a national database of EMS information in the United States) by comparing patient ages as documented in NEMSIS to the ages of all ED arrivals documented in other sources (the results showed high correlation, *r* > 0.9).[41] Lerner et al*.* (2021) evaluated a pediatric-specific database with the complete cohort of all pediatric records in NEMSIS and found meaningful differences in patient race and chief complaints between the two groups [69].

Other linkage studies assessed their results for bias by examining differences between linked and unlinked cohorts. Within particular clinical areas, such as stroke and OHCA, indications of bias between linked and unlinked groups were seen within topic-related factors, such as age, event location, bystander CPR, or return of spontaneous circulation [57, 68, 70, 71] . Another study linking paramedic and hospital records tracked the degree to which an optimized strategy for case matching mitigated bias found in a standard approach [19].

### Quality thresholds

Also included as an attribute of the nature of studies on research in paramedicine, the concept of quality thresholds appeared sporadically among the included studies. Despite these mentions, there are no established guides, thresholds, or systems for defining what constitutes quality data or determining what is high versus low quality. Many studies discussed the relevance of their results, finding them to be feasible or applicable (or not) in individual cases. Few studies reported applying any quality threshold; those that did are described below.

The domain of completeness offered clear and simple options for testing. In one study, completeness of less than 90% (or greater than 10% missingness) was judged to be low quality [36]. Others used similar thresholds [45, 72–74]. Within studies examining linkage of paramedic data with other sources, papers sometimes applied a pre-specified probability cut-off that determined a match or non-match, with those at or near the threshold value being selected for manual review. This was often listed as a probability at or straddling 0.9, [39, 51, 75] although 0.5 was also used, [49] as were levels that varied within the study according to patient block [53]. Other studies used ratings of match quality depending on the number or type of variables that established the link [70, 76, 77].

Within the domain of representativeness, few studies worked with a standard beyond reporting different proportions among their study groups. In contrast, Lerner et al*.* (2021) described applying a threshold of plus or minus 5% as indicating a meaningful difference between their sample and reference populations [69]. Oostema et al*.* used an absolute standardized difference, defined as the average difference of each variable as a percent of its standard deviation, with values greater than 0.1 indicating a significant difference [68].

## Discussion

The studies identified in this scoping review make up a sample of DQA practices in research in paramedicine. This collection varies widely across many factors, including country of origin, topic assessed, and purpose. In many cases, the DQA component appeared to be ad hoc, reflecting the unique methodological requirements of individual studies and often presented as an accompanying abstract or article to an investigation with some other aim. Where evident, accumulated expertise developed over the course of multiple studies appeared within related research groups, rather than across researchers within the profession [38, 39, 51, 69, 75, 78]. The variety in purpose was also related to the extent of included studies. Many featured a single service examining its own data or linking to a single hospital or ED. In contrast, there were several examples of regional, state, or national-level data being integrated with multiple external databases with high levels of linkage success, either for specific research purposes or routine outcome evaluation [19, 34, 51, 70, 79]. These examples demonstrate progress in overcoming oft-noted barriers to data linkage and outcome evaluation [2, 11].

While the results of individual studies were too variable to draw specific conclusions about paramedic data quality, some generalizations about the nature of DQA practices emerged. Many authors emphasized the central priority of data completeness in paramedic research. Although a relatively simple concept, completeness was seen as a foundation supporting other domains – not only as a baseline indicator of data quality, but also as an essential precursor to linkage with other databases and outcome evaluation. Apart from this consensus, there were few (if any) common standards in terms of variables, domains, methods, or quality thresholds for DQA in paramedic research. A DQA framework was mentioned by only one included study (which was only partially applicable to prehospital data) [33]. Relatedly, although a reporting guideline exists for data-linkage methodology, it was referenced by only two papers out of 34 reporting linkage results [34, 35]. As in existing frameworks, the terminology and application of some DQA practices among the included studies featured variable or inconsistent meanings. This variety highlights the need for clear and consistent terminology to support transparency and comparability in DQA practices.

These characteristics of DQA practices point to both the relative youth of research in paramedicine and continuing barriers to research and data collection in the field in general [9, 10]. These barriers are discussed at length by several articles, and key findings reiterate the difficulty of collecting high-quality information (especially accurate demographic details) in the clinical environment [36]. Incomplete or unreliable data limit the effectiveness of deterministic linkage, [52] and inconsistent reporting of common data fields complicates studies using aggregated data. Problems with varied reporting were observed among a range of topic areas, including defining trauma calls, [52] coding chief complaints, [38] reporting OHCA variables, [67] and even the ages defining pediatric patients, which ranged among included studies from 0–4 to 0–21 [69, 80–82]. These inconsistencies overlapped with observed difficulties in both coding and extracting information from free-text data [38, 83]. Data linkage is complex, labour-intensive, and expensive, presenting challenges to single services aiming to assess outcomes [70]. Finally, the need to establish data-sharing agreements between organizations that collaborate in patient care constitutes another barrier to outcome assessment [52].

Although challenges to data quality were widely described, fewer studies remarked on strategies for assurance or improvement. Among those that did, Mann et al*.* referenced a system of over 300 logic rules that assess data quality prior to acceptance in NEMSIS [41]. (While logic rules are commonly applied, one paper observed the unintended consequence of a “bare minimum effect” when forcing documentation [36].) Several studies showed improved documentation after focused and dedicated internal training [83–85]. Others noted improved outcomes with the introduction of electronic forms or databases [86–88]. Methodological refinements in case ascertainment, handling missing data, and linkage strategies were shown to maximize data quality [39, 51, 75].

Beyond the barriers and strategies for improvement for data quality in general, the included studies speak to DQA practices both by what they describe and by what they do not. Existing DQA frameworks feature domains and sub-domains that did not appear among the reviewed studies, including broad categories such as accessibility, clarity, and timeliness [32, 89] These domains (as well as synonyms and related concepts such as punctuality, relevance, interpretability, comparability) largely reflect the needs of researchers in gaining access to databases, the timing of data updates and their availability, and supporting documentation [14, 32, 89] (Occasional studies have assessed the timeliness of the availability of the paramedic record for clinical use, but not for research purposes [90, 91]) The absence of these domains might be seen also to reflect the relative youth of paramedic databases and remaining barriers to incorporating them into administrative repositories.

Considering DQA along a spectrum of progress highlights current issues and how they might be incorporated into the next iterations of guidelines for paramedic data. As an example, recent research has foregrounded comprehensive reporting of sex and gender and the inadequacy of binary options to encompass multi-dimensional concepts [92]. Sex and gender reporting has been evaluated in other electronic health datasets, [93] and the implications of its limitations on record linkage were considered in one included study [94]. In a similar approach, the COVID-19 pandemic has accelerated efforts to examine outcomes through the lens of data equity, [95] and current guidance on race-based data collection emphasizes a range of system features that might be considered preconditions for the responsible collection and use of this information [96]. Finally, knowledge of patient and public perspectives related to individual data items translates to awareness of public involvement and engagement in data management as a precursor to maintaining social license for healthcare research [97, 98]. While concepts such as data ownership, stewardship, and patient and public involvement do not address quality in the same way as ensuring birthdates are collected accurately, they undoubtedly have a role in how data is collected, accessed, and used – and therefore a role in ensuring the most basic definition of data quality, that it is fit for use [32].

## Limitations

While comprehensive, the search strategy employed in this review was necessarily exploratory. It was iteratively refined to ensure capture of known key papers, but the possibility of missed articles cannot be excluded, and the resulting sample could be biased in unknown ways. Extreme heterogeneity among included studies presents difficulty in summarizing results. Alternative ways of categorizing and interpreting the data are possible, and the approach taken here potentially reflects biases among the reviewers. Although small, the review team included members with clinical, administrative, and methodological expertise in order to guard against this possibility. In keeping with the nature of scoping reviews, these results should be taken as a preliminary description of the field of study, with analyses and conclusions interpreted cautiously.

## Conclusions

This scoping review of DQA practices in paramedic research summarizes diverse approaches applied largely as needed in individual studies or research programs. Although there are many opportunities and options for improving the quality of data collected at the source, the results of this review point to additional considerations for practice leaders. Databases of health information collected by paramedics would benefit from a standardized framework for DQA that allows for local variation while establishing common methods, terminology, and reporting standards. As paramedic research continues to grow, there is an opportunity to integrate progressive concepts of availability, stewardship, and ownership into emerging constructs.

### Supplementary Information

**Additional file 1 ** Documentation of searches.

**Additional file 2 ** Data extraction form.

**Additional file 3 ** Citations to all articles included in the review.

## Data Availability

All source material is available in the public domain. Translations of included articles not in English are available upon reasonable request.

## Abbreviations

DQA: Data quality assessment
PRISMA-ScR: Preferred Reporting Items in Systematic Reviews and Meta-analyses extension for scoping reviews
CIHI: Canadian Institute for Health Information
OHCA: Out-of-hospital cardiac arrest
ED: Emergency department
EMS: Emergency medical services
NEMSIS: National Emergency Medical Services Information System

## Footnotes

1 * Other common descriptions of paramedicine and paramedics reflect distinct aspects of the profession, whether in terms of the provider (emergency medical technicians, emergency responder, allied health provider) or the setting (prehospital care, out-of-hospital care, remote and retrieval medicine). No single description encompasses the international range of past and emerging practice models. Acknowledging this limitation, this review will use “paramedic” and “paramedicine” as generally inclusive of varied roles and settings.
