# Supplementary material for: Database quality assessment in research in paramedicine: a scoping review
Source: Scand J Trauma Resusc Emerg Med. 2023 Nov 11;31:78. doi: 10.1186/s13049-023-01145-2 (PMC10638787; doi:10.1186/s13049-023-01145-2)
Supplement: Supplementary file 1 — Additional file 1 Documentation of searches. [file 13049_2023_1145_MOESM1_ESM.pdf]

Additional File 1: Search strategies

MEDLINE

1 emergency medical services.sh.
2 Emergency Medical Technicians/st, sn [Standards, Statistics & Numerical Data]
3 Ambulances/st, sn [Standards, Statistics & Numerical Data]
4 paramed$.tw.
5 prehospital.tw.
6 pre-hospital.tw.
7 ambulance.tw.
8 ems.tw.
9 emt.tw.
10 "first respond$".tw.
11 "emergency medical technician$".tw.
12 "emergency services".tw.
13 1 or 2 or 3 or 4 or 5 or 6 or 7 or 8 or 9 or 10 or 11 or 12
14 Quality Improvement/st, sn, td [Standards, Statistics & Numerical Data, Trends]
15 Quality Assurance, Health Care/mt, st, sn, td [Methods, Standards, Statistics & Numerical Data, Trends]
16 "Information Storage and Retrieval"/mt, st, sn, td [Methods, Standards, Statistics & Numerical Data, Trends]
17 Data Collection/
18 Medical Records/
19 Electronic Health Records/
20 Health Records, Personal/
21 Medical Record Linkage/
22 Medical Records Systems, Computerized/
23 Registries/mt, og, st, sn [Methods, Organization & Administration, Standards, Statistics & Numerical Data]
24 (data$ adj3 (quality, or link$ or accu$ or digit$ or electronic$ or record or paramed$ or prehospital or pre-hospital)).tw.
25 "electronic medical record".tw.
26 "record linkage".tw.
27 "paramedic record".tw.
28 14 or 15 or 16 or 17 or 18 or 19 or 20 or 21 or 22 or 23 or 24 or 25 or 26 or 27
29 13 and 28
30 limit 29 to yr="2011 -Current"

EMBASE

1 emergency health service/
2 rescue personnel/
3 ambulance transportation/
4 ambulance/
5 paramed$.tw.
6 prehospital.tw.
7 pre-hospital.tw.
8 ambulance.tw.
9 ems.tw.
10 "first respond$".tw.
11 "emergency medical technician$".tw.
12 "emergency services".tw.
13 1 or 2 or 3 or 4 or 5 or 6 or 7 or 8 or 9 or 10 or 11 or 12
14 total quality management/
15 *clinical effectiveness/ or *performance measurement system/ or *program evaluation/ or *public health systems research/
16 information retrieval/
17 information storage/
18 data extraction/
19 medical information system/
20 electronic medical record/ or electronic medical record system/
21 *electronic health record/ or *electronic patient record/
22 *patient registry/ or *clinical trial registry/ or *death registry/ or *disease registry/
23 (data$ adj3 (quality, or link$ or accu$ or digit$ or electronic$ or record or paramed$ or prehospital or pre-hospital)).tw.
24 "electronic medical record".tw.
25 "record linkage".tw.
26 "paramedic record".tw.
27 14 or 15 or 16 or 17 or 18 or 19 or 20 or 21 or 22 or 23 or 24 or 25 or 26
28 13 and 27
29 limit 28 to yr="2011 -Current"

Scopus

1	KEY "emergency medical services"
2	KEY "emergency medical technicians"
3	KEY "ambulance"
4	KEY paramedic
5	KEY prehospital
6	KEY pre-hospital
7	KEY "first respond*"
8	KEY "emergency services"
9	1 OR 2 OR 3 OR 4 OR 5 OR 6 OR 7 OR 8 OR 9
10	KEY "quality improvement"
11	KEY “quality assurance, health care"
12	KEY "information storage"
13	KEY "information retrieval"
14	KEY "data collection"
15	KEY "medical records"
16	KEY "electronic health records"
17	KEY "health records, personal"
18	KEY "medical record linkage"
19	KEY "medical records systems, computerized"
20	KEY "patient regist*"
21	TITLE-ABS-KEY data* W/3 ("quality" OR "link*" OR "accu*" OR "digit*" OR "electronic*" OR "record" OR "paramedic" OR "prehospital" OR "pre-hospital")
22	TITLE-ABS-KEY "electronic medical record"
23	TITLE-ABS-KEY "record linkage"
24	TITLE-ABS-KEY "paramedic record"
25	10 OR 11 OR 12 OR 13 OR 14 OR 15 OR 16 OR 17 OR 18 O 19 OR 20 OR 21 OR 22 OR 23 OR 24
26	9 AND 25
27	LIMIT 26 (2011 - CURRENT)

CINAHL

1	(MH "Emergency Medical Services")	Expanders - Apply equivalent subjects
2	(MH "Emergency Medical Technicians")	Expanders - Apply equivalent subjects
3	(MH "Ambulances")	Expanders - Apply equivalent subjects
4	TX paramedic	Expanders - Apply equivalent subjects
5	TX prehospital	Expanders - Apply equivalent subjects
6	TX pre-hospital	Expanders - Apply equivalent subjects
7	TX ambulance	Expanders - Apply equivalent subjects
8	TX ems	Expanders - Apply equivalent subjects
9	TX emt	Expanders - Apply equivalent subjects
10	TX "first respond#"	Expanders - Apply equivalent subjects
11	TX "emergency medical technician#"	Expanders - Apply equivalent subjects
12	TX "emergency services"	Expanders - Apply equivalent subjects
**13**	**S1 OR S2 OR S3 OR S4 OR S5 OR S6 OR S7 OR S8 OR S9 OR S10 OR S11 OR S12**	**Expanders - Apply equivalent subjects**
14	(MH "Quality Improvement/TD/SN/ST")	Expanders - Apply equivalent subjects
15	(MH "Quality of Health Care/TD/SN/ST/MT")	Expanders - Apply equivalent subjects
16	(MH "Information Retrieval/MT/ST/TD/EV")	Expanders - Apply equivalent subjects
17	(MH "Information Storage/EV/MT/ST/TD")	Expanders - Apply equivalent subjects
18	(MH "Data Collection")	Expanders - Apply equivalent subjects
19	(MH "Medical Records")	Expanders - Apply equivalent subjects
20	(MH "Electronic Health Records")	Expanders - Apply equivalent subjects
21	(MH "Medical Records, Personal")	Expanders - Apply equivalent subjects
22	(MH "Medical Record Linkage")	Expanders - Apply equivalent subjects
23	(MH "Registry Personnel/MT/ST/SN/TD")	Expanders - Apply equivalent subjects
24	TX data# N3 (quality or link# or accu# or digit# or electronic# or record or paramedic or prehospital or pre-hospital)	Expanders - Apply equivalent subjects
25	TX "electronic medical record"	Expanders - Apply equivalent subjects
26	TX "record linkage"	Expanders - Apply equivalent subjects
27	TX "paramedic record"	Expanders - Apply equivalent subjects
**28**	**S14 OR S15 OR S16 OR S17 OR S18 OR S19 OR S20 OR S21 OR S22 OR S23 OR S24 OR S25 OR S26 OR S27**	**Expanders - Apply equivalent subjects**
**29**	**S13 AND S28**	**Limiters - Published Date: 20110101-20211231**
