# Supplementary material for: Database quality assessment in research in paramedicine: a scoping review
Source: Scand J Trauma Resusc Emerg Med. 2023 Nov 11;31:78. doi: 10.1186/s13049-023-01145-2 (PMC10638787; doi:10.1186/s13049-023-01145-2)
Supplement: Supplementary file 2 — Additional file 2 Data extraction form. [file 13049_2023_1145_MOESM2_ESM.pdf]

Additional File 2: Data-extraction form

	RANGE				EXTENT			NATURE					
**Study**	**Location**	**Year**	**Purpose**	**Clinical area**	**Level of Data Being Assessed**	**Breadth of Data Being Assessed**	**Number of records assessed**	**Prehospital Data Field Assessed**	**Method of Assessment**	**Result of Assessment**	**Domain - as identified by study, if done**	**CIHI domain, if applicable**	**Quality threshold**
Study citation	*Geographic location of data being assessed*	*Of publication*	*As stated by study*	*If applicable*	*Refers to jurisdictional level, whether individual service, state / province, national, international*	*Refers to spread of data being assessed at whichever level: for example, data from one service linked to multiple state / provincial databases*		*Variables assessed. If possible, these will be summarized by general categories, such as: Patient demographics, call characteristics, subjective information, interventions, vital signs, etc.*	*Can include overall research method, experimental design, and specific assessment techniques*	*As reported, potentially summarized by category*			*Any threshold or measure used to judge quality*

CIHI: Canadian Institute for Health Information
