# Supplementary material for: Database quality assessment in research in paramedicine: a scoping review
Source: Scand J Trauma Resusc Emerg Med. 2023 Nov 11;31:78. doi: 10.1186/s13049-023-01145-2 (PMC10638787; doi:10.1186/s13049-023-01145-2)
Supplement: Supplementary file 3 — Additional file 3 Citations to all articles included in the review. [file 13049_2023_1145_MOESM3_ESM.pdf]

Additional File 3: All included studies

Abir M, Taymour RK, Goldstick JE, et al. Data missingness in the Michigan NEMSIS (MI-EMSIS) dataset: a mixed-methods study. *Int J Emerg Med*. 2021;14(1):22. doi:https://dx.doi.org/10.1186/s12245-021-00343-y

Alstrup K, Petersen JAK, Barfod C, Knudsen L, Rognås L, Møller TP. The Danish helicopter emergency medical service database: high quality data with great potential. *Scand J Trauma Resusc Emerg Med*. Apr 5 2019;27(1):38. doi:10.1186/s13049-019-0615-5

Andrews R, Wynn MT, Ter Hofstede AHM, et al. Leveraging data quality to better prepare for process mining: An approach illustrated through analysing road trauma pre-hospital retrieval and transport processes in Queensland. *Int J Environ Res Public Health*. 2019;16(7):1138. doi:http://dx.doi.org/10.3390/ijerph16071138

Andrusiek DL, Hall CA, Votova KM, Randhawa GK. Use of force in police-public encounters and medical outcomes: Issues with linking police and emergency medical services (EMS) data. *CJEM*. 2012;14(SUPPL. 1):S49. 2012 CAEP/ACMU Scientific Abstracts. Niagara Falls, ON Canada.

Asimos AW, Ward S, Brice JH, et al. A geographic information system analysis of the impact of a statewide acute stroke emergency medical services routing protocol on community hospital bypass. *J Stroke Cerebrovasc Dis*. 2014;23(10):2800-2808. doi:http://dx.doi.org/10.1016/j.jstrokecerebrovasdis.2014.07.004

Babcock L, Lloyd J, Semenova O, Meinzen-Derr J, Depinet H. Prehospital capture of variables commonly used in ED sepsis screening tools. *Pediatrics*. 2019;144(2)National Conference on Education 2018. Orlando, FL United States. doi:http://dx.doi.org/10.1542/peds.144.2-MeetingAbstract.412

Barley CR, Gunson IM. Rates of recording different aspects of patients' social history on ambulance electronic patient records-a service evaluation. *Emerg Med J*. 2021;38(9)999 EMS Research Forum 2021 Conference. Online. doi:http://dx.doi.org/10.1136/emermed-2021-999.38

Berben SAA, Scholten AC, Westmaas AH, et al. Pain management in trauma patients in (pre)hospital based emergency care: Current practice versus new guideline. *Injury*. 2015;46(5):798-806. doi:http://dx.doi.org/10.1016/j.injury.2014.10.045

Bergrath S, Skorning M, RÃ¶rtgen D, et al. Is paper-based documentation in an emergency medical service adequate for retrospective scientific analysis? An evaluation of a physician-run service. *Emerg Med J*. 2011;28(4):320-324. doi:10.1136/emj.2009.086538

Bessant G, Dharmaratne S. Annual tourniquet use in UK ambulance services for major haemorrhage control. *BMJ Open*. 2017;7(Supplement 3):A5. 2nd European Emergency Medical Services Congress, EMS 2017. Copenhagen Denmark. doi:http://dx.doi.org/10.1136/bmjopen-2017-EMSabstracts.13

Betlehem J, Deutsch K, Marton J, et al. The importance of accurate examination of stroke patients in prehospital emergency care. *Cerebrovascular Diseases*. 2013;35(SUPPL. 3):478. 22nd European Stroke Conference. London United Kingdom.doi:http://dx.doi.org/10.1159/000353129

Blanchard IE, Williamson TS, Ronksley P, et al. Linkage of Emergency Medical Services and Hospital Data: A Necessary Precursor to Improve Understanding of Outcomes of Prehospital Care. *Prehosp Emerg Care*. Oct 20 2021:1-10. doi:10.1080/10903127.2021.1977438

Bloomer R, Burns BJ, Ware S. Improving documentation in prehospital rapid sequence intubation: investigating the use of a dedicated airway registry form. *Emerg Med J*. 2013;30(4):324-6. doi:https://dx.doi.org/10.1136/emermed-2011-200715

Bradley NL, Garraway N, Bell N, Lakha N, Hameed SM. Data capture and communication during transfers to definitive care in an inclusive trauma system. *Injury*. 2017;48(5):1069-1073. doi:https://dx.doi.org/10.1016/j.injury.2016.11.004

Carroll T, Muecke S, Simpson J, Irvine K, Jenkins A. Quantification of NSW Ambulance Record Linkages with Multiple External Datasets. *Prehosp Emerg Care*. 2015;19(4):504-15. doi:https://dx.doi.org/10.3109/10903127.2015.1025154

Chikani V, Blust R, Vossbrink A, et al. Improving the Continuum of Care by Bridging the Gap between Prehospital and Hospital Discharge Data through Stepwise Deterministic Linkage. *Prehosp Emerg Care*. 2020;24(1):1-7. doi:https://dx.doi.org/10.1080/10903127.2019.1604925

Clark SJ, Halter M, Porter A, et al. Using deterministic record linkage to link ambulance and emergency department data: is it possible without patient identifiers? A case study from the UK. *Int J Popul Data Sci*. 2019;4(1):1104. doi:https://dx.doi.org/10.23889/ijpds.v4i1.1104

Coventry LL, Bremner AP, Williams TA, Jacobs IG, Finn J. Symptoms of myocardial infarction: concordance between paramedic and hospital records. *Prehosp Emerg Care*. 2014;18(3):393-401. doi:https://dx.doi.org/10.3109/10903127.2014.891064

Cox S, Martin R, Somaia P, Smith K. The development of a data-matching algorithm to define the 'case patient'. *Aust Health Rev*. 2013;37(1):54-9. doi:https://dx.doi.org/10.1071/AH11161

Crilly JL, O'Dwyer JA, O'Dwyer MA, et al. Linking ambulance, emergency department and hospital admissions data: understanding the emergency journey. *Med J Aust*. Feb 21 2011;194(4):S34-7.

Cunningham J, Williamson D, Robinson KM, Carroll R, Buchanan R, Paul L. The quality of medical record documentation and External cause of fall injury coding in a tertiary teaching hospital. *Health Inf Manag*. 2014;43(1):6-15.

Deasy C, Hall D, Bray JE, Smith K, Bernard SA, Cameron P. Paediatric out-of-hospital cardiac arrests in Melbourne, Australia: improved reporting by adding coronial data to a cardiac arrest registry. *Emerg Med J*. 2013;30(9):740-744. doi:10.1136/emermed-2012-201531

Demel SL, Nickles AV, O'Brien S, et al. Documentation of last known well time in the Michigan stroke coverdell registry. *Stroke*. 2018;49(Supplement 1)American Heart Association/American Stroke Association 2018 International Stroke Conference and State-of-the-Science Stroke Nursing Symposium. Los Angeles, CA United States.

Depinet HE, Eckerle M, Semenova O, Meinzen-Derr J, Babcock L. Characterization of Children with Septic Shock Cared for by Emergency Medical Services. *Prehosp Emerg Care*. 2019;23(4):491-500. doi:https://dx.doi.org/10.1080/10903127.2018.1539147

Dewolf P, Rutten B, Wauters L, et al. Impact of video-recording on patient outcome and data collection in out-of-hospital cardiac arrests. *Resuscitation*. 2021;165:1-7. doi:https://dx.doi.org/10.1016/j.resuscitation.2021.05.033

Engels PT, Coates A, MacDonald RD, et al. Toward an all-inclusive trauma system in Central South Ontario: development of the Trauma-System Performance Improvement and Knowledge Exchange (T-SPIKE) project. *Can J Surg*. 2021;64(2):E162-E172. doi:https://dx.doi.org/10.1503/cjs.000820

Fein M, Quinn J, Watt K, Nichols T, Kimble R, Cuttle L. Prehospital paediatric burn care: New priorities in paramedic reporting. *Emerg Med Australas*. 2014;26(6):609-15. doi:https://dx.doi.org/10.1111/1742-6723.12313

Fix J, Ising AI, Proescholdbell SK, et al. Linking Emergency Medical Services and Emergency Department Data to Improve Overdose Surveillance in North Carolina. *Public Health Rep*. Nov-Dec 2021;136(1_suppl):54s-61s. doi:10.1177/00333549211012400

Fosbol EL, Granger CB, Peterson ED, et al. Prehospital system delay in ST-segment elevation myocardial infarction care: A novel linkage of emergency medicine services and inhospital registry data. *Am Heart J*. 2013;165(3):363-370. doi:10.1016/j.ahj.2012.11.003

Foster A, Florea V, Fahrenbruch C, Blackwood J, Rea TD. Availability and Accuracy of EMS Information about Chronic Health and Medications in Cardiac Arrest. *West J Emerg Med*. 2017;18(5):864-869. doi:10.5811/westjem.2017.5.33198

Frisch A, Reynolds JC, Condle J, Gruen D, Callaway CW. Documentation discrepancies of time-dependent critical events in out of hospital cardiac arrest. *Resuscitation*. 2014;85(8):1111-4. doi:https://dx.doi.org/10.1016/j.resuscitation.2014.05.002

Gaeeni M, Vanosfaderani MR, Masoud MP, Hamta A. [Comparison of Time Indicators and Outcome of Pre-hospital Emergency Operations in Two Methods of Electronic Registration with Asayar Program and Paper Registration.] *Qom University of Medical Sciences Journal*. 2021;14(12):22-31. doi:10.29252/qums.14.12.22

Garcia Minguito L, Casas Sanchez JdD, Rodriguez Albarran MS. [A proposed scale to analyze the quality of injury reports in cases of gender violence]. *Propuesta de baremo (de escala) para analizar la calidad de los partes de lesiones en casos de violencia de genero*. 2012;26(3):256-60. doi:https://dx.doi.org/10.1016/j.gaceta.2011.07.025

Gerhardt RT, Reeves PT, Kotwal RS, Mabry RL, Robinson JB, Butler F. Analysis of Prehospital Documentation of Injury-Related Pain Assessment and Analgesic Administration on the Contemporary Battlefield. *Prehosp Emerg Care*. 2016;20(1):37-44. doi:10.3109/10903127.2015.1051683

Goldstick J, Ballesteros A, Flannagan C, Roche J, Schmidt C, Cunningham RM. Michigan system for opioid overdose surveillance. *Inj Prev*. 2021;doi:https://dx.doi.org/10.1136/injuryprev-2020-043882

Govindarajan P, Mobed K, Johnston C, Ghilarducci D. Probabilistic linkage of emergency medical services records and statewide emergency and patient discharge data. *Acad Emerg Med*. 2011;18(5 SUPPL. 1):S118. 2011 Annual Meeting of the Society for Academic Emergency Medicine, SAEM. Boston, MA United States.doi:http://dx.doi.org/10.1111/j.1553-2712.2011.01073.x

Gravens B, Pistey M, McNett M, Reed E, Wilson LD, Piktel JS. Use of electronic health records to identify cardiac disease substrates during resuscitation from cardiac arrest. *Circulation*. 2018;138(25):e779. American Heart Association's Scientific Sessions 2018 and Resuscitation Science Symposium. Chicago, IL United States. doi:http://dx.doi.org/10.1161/CIR.0000000000000636

Halbesma N, Clarke S, Clegg G, Bywater D, Bijman L, Lynch E. Linking pre-hospital out-of-hospital cardiac arrest data to in-hospital outcomes in order to improve the 'chain of survival'. *Emerg Med J*. 2019;36(1):E9-E10. 999 EMS Research Forum Conference 2018. Stirling United Kingdom. doi:http://dx.doi.org/10.1136/emermed-2019-999.23

Hern HG, Alter H, Barger J, Teves M, Hamilton K, Mueller L. A focused educational intervention increases paramedic documentation of patient pain complaints. *Acad Emerg Med*. 2012;19(SUPPL. 1):S202-S203. 2012 Annual Meeting of the Society for Academic Emergency Medicine, SAEM 2012. Chicago, IL United States. doi:http://dx.doi.org/10.1111/j.1553-2712.2012.01332.x

Hu P, Galvagno Jr SM, Jordan S, et al. Identification of dynamic prehospital changes with continuous vital signs acquisition. *Air Med J*. 2014;33(1):27-33. doi:http://dx.doi.org/10.1016/j.amj.2013.09.003

Hughes-Gooding T, Dickson JM, O'Keeffe C, Mason SM. A data linkage study of suspected seizures in the urgent and emergency care system in the UK. *Emerg Med J*. 2020;37(10):605-610. doi:https://dx.doi.org/10.1136/emermed-2019-208820

Ibrahim G, Nickles AV, Wall SR, et al. Assessing the accuracy of a linkage between the michigan emergency medical services information system and the michigan coverdell acute stroke registry. *Stroke*. 2019;50(Supplement 1)American Heart Association/American Stroke Association 2019 International Stroke Conference and State-of-the-Science Stroke Nursing Symposium. Honolulu, HI United States. doi:http://dx.doi.org/10.1161/str.50.suppl_1.WP316

Jaureguibeitia X, Aramendi E, Irusta U, et al. Methodology and framework for the analysis of cardiopulmonary resuscitation quality in large and heterogeneous cardiac arrest datasets. *Resuscitation*. Nov 2021;168:44-51. doi:10.1016/j.resuscitation.2021.09.005

Ji C, Quinn T, Gavalova L, et al. Feasibility of data linkage in the PARAMEDIC trial: a cluster randomised trial of mechanical chest compression in out-of-hospital cardiac arrest. *BMJ Open*. Jul 28 2018;8(7):e021519. doi:10.1136/bmjopen-2018-021519

Katzer R, Barton DJ, Adelman S, Clark S, Seaman EL, Hudson KB. Impact of implementing an EMR on physical exam documentation by ambulance personnel. *Applied clinical informatics*. 2012;3(3):301-8. doi:https://dx.doi.org/10.4338/ACI-2012-03-RA-0008

Kearney AS, George N, Karim N, et al. Development of a trauma and emergency database in Kigali, Rwanda. *Afr J Emerg Med*. 2016;6(4):185-190. Developpement d'une base de donnees sur les traumatismes et les urgences a Kigali, Rwanda. doi:http://dx.doi.org/10.1016/j.afjem.2016.10.002

Ko PC-I, Chiang W-C, Ma MH-M, et al. Innovative Web-based e-registry enhances survival after out-of-hospital cardiac arrest. *Circulation*. 2012;126(21 SUPPL. 1)American Heart Association 2012 Scientific Sessions and Resuscitation Science Symposium. Los Angeles, CA United States.

Kummer B, Mehendale R, Williams O, et al. Clinical information systems integration in New York city's first mobile stroke unit. *European Stroke Journal*. 2017;2(1 Supplement 1):243. 3rd European Stroke Organisation Conference, ESOC 2017. Prague Czechia. doi:http://dx.doi.org/10.1177/2396987317705242

Lerner EB, Dayan PS, Brown K, et al. Characteristics of the pediatric patients treated by the Pediatric Emergency Care Applied Research Network's affiliated EMS agencies. *Prehosp Emerg Care*. 2014;18(1):52-9. doi:https://dx.doi.org/10.3109/10903127.2013.836262

Lerner EB, Browne LR, Studnek J, et al. Novel use of the National Emergency Medical Services Information System to create a pediatric emergency care applied research network-specific emergency medical services

patient registry. *Academic Emergency Medicine*. 2021;28(SUPPL 1):S113. Society for Academic Emergency Medicine Annual Meeting, SAEM 2021. Virtual. doi:http://dx.doi.org/10.1111/acem.14249

Li T, Zhu N, Jones CMC, Shah MN. Accuracy of medical history and medications documented by emergency medical services. *Acad Emerg Med*. 2016;23(SUPPL. 1):S254-S255. 2016 Annual Meeting of the Society for Academic Emergency Medicine, SAEM 2016. New Orleans, LA United States. doi:http://dx.doi.org/10.1111/acem.12974

Lippert F, Folke F, Christensen HC, Blomberg SN. Transition of medical records from paper to electronic records - implications for out-of-hospital cardiac arrest registration. *Resuscitation*. 2019;142(Supplement 1):e78. RESUSCITATION 2019 - Controversies in Resuscitation. Ljubljana Slovenia. doi:http://dx.doi.org/10.1016/j.resuscitation.2019.06.187

MacDougall L, Smolina K, Otterstatter M, et al. Development and characteristics of the Provincial Overdose Cohort in British Columbia, Canada. *PloS one*. 2019;14(1):e0210129. doi:https://dx.doi.org/10.1371/journal.pone.0210129

Mann NC, Kane L, Dai M, Jacobson K. Description of the 2012 NEMSIS public-release research dataset. *Prehosp Emerg Care*. Apr-Jun 2015;19(2):232-40. doi:10.3109/10903127.2014.959219

McDonald S, Fowler R, Owens P, May S, Herren H, Idris AH. Automating medical record matching: A key component of an automated cardiac arrest registry. *Circulation*. 2020;140(Supplement 2)American Heart Association's 2019 Resuscitation Science Symposium, ReSS 2019. Philadelphia, PA United States. doi:http://dx.doi.org/10.1161/circ.140.suppl_2.238

Miller ML, Lincoln EW, Brown LH. Development of a Binary End-of-Event Outcome Indicator for the NEMSIS Public Release Research Dataset. *Prehosp Emerg Care*. 2021;25(4):504-511. Erratum in: Prehosp Emerg Care. 2021 Jan 19;:1; PMID: 33464937 [https://www.ncbi.nlm.nih.gov/pubmed/33464937]. doi:https://dx.doi.org/10.1080/10903127.2020.1794435

Mumma BE, Diercks DB, Danielsen B, Holmes JF. Probabilistic Linkage of Prehospital and Outcomes Data in Out-of-hospital Cardiac Arrest. *Prehosp Emerg Care*. 2015 Jul-Sep 2015;19(3):358-64. doi:10.3109/10903127.2014.980474

Newgard CD, Zive D, Malveau S, Leopold R, Worrall W, Sahni R. Developing a statewide emergency medical services database linked to hospital outcomes: a feasibility study. *Prehosp Emerg Care*. 2011;15(3):303-19. doi:https://dx.doi.org/10.3109/10903127.2011.561404

Mysliwiec R, Clark S, Bloemen EM, Stern M, Flomenbaum N. Descriptive analyses of prehospital documentation for older adults presenting to the emergency department. *J Am Geriatr Soc*. 2015;63(SUPPL. 1):S37. 2015 Annual Scientific Meeting of the American Geriatrics Society. National Harbor, MD United States.doi:http://dx.doi.org/10.1111/jgs.13439

Newgard C, Malveau S, Staudenmayer K, et al. Evaluating the use of existing data sources, probabilistic linkage, and multiple imputation to build population-based injury databases across phases of trauma care. *Acad Emerg Med*. 2012;19(4):469-480. doi:http://dx.doi.org/10.1111/j.1553-2712.2012.01324.x

Newgard CD, Malveau S, Zive D, Lupton J, Lin A. Building A Longitudinal Cohort From 9-1-1 to 1-Year Using Existing Data Sources, Probabilistic Linkage, and Multiple Imputation: A Validation Study. *Acad Emerg Med*. 2018;25(11):1268-1283. doi:https://dx.doi.org/10.1111/acem.13512

Newgard CD, Zive D, Weathers C, Jui J, Daya M. Electronic versus manual data processing: Evaluating the use of electronic health records in out-of-hospital clinical research. *Academic Emergency Medicine*. 2012;19(2):217-227. doi:http://dx.doi.org/10.1111/j.1553-2712.2011.01275.x

Nishiyama C, Brown SP, May S, et al. Apples to apples or apples to oranges? International variation in reporting of process and outcome of care for out-of-hospital cardiac arrest. *Resuscitation*. 2014;85(11):1599-1609. doi:10.1016/j.resuscitation.2014.06.031

Oostema JA, Nickles A, Reeves MJ. A Comparison of Probabilistic and Deterministic Match Strategies for Linking Prehospital and in-Hospital Stroke Registry Data. *J Stroke Cerebrovasc Dis*. Oct 2020;29(10):105151. doi:10.1016/j.jstrokecerebrovasdis.2020.105151

Oud FRW, Kooij FO, Burns BJ. Long-term Effectiveness of the Airway Registry at Sydney Helicopter Emergency Medical Service. *Air Med J*. 2019;38(3):161-164. doi:10.1016/j.amj.2019.01.006

Outterson S, Jaque A, Frisch A, et al. Prehospital and in-hospital chart agreement for patients with chest pain. *Acad Emerg Med*. 2016;23(SUPPL. 1):S128-S129. 2016 Annual Meeting of the Society for Academic Emergency Medicine, SAEM 2016. New Orleans, LA United States. doi:http://dx.doi.org/10.1111/acem.12974

Perez O, Barnhart BJ, Hu C, et al. Prehospital blood pressure measurement in major traumatic brain injury: Concordance between EMS provider documentation and non-invasive monitor data tracking. *Circulation*. 2017;136(Supplement 1)Resuscitation Science Symposium, ReSS 2017. Anaheim, CA United States.

Perez O, Barnhart BJ, Spaite DW, et al. Accuracy of ems hypoxia documentation compared to continuous non-invasive monitor data in major traumatic brain injury. *J Emerg Med*. 2017;53(3):443. 9th Mediterranean Emergency Medicine Congress, MEMC 2017. Lisbon Portugal. doi:http://dx.doi.org/10.1016/j.jemermed.2017.08.063

Poulsen NR, Klojgard TA, Lubcke K, Lindskou TA, Sovso MB, Christensen EF. Completeness in the recording of vital signs in ambulances increases over time. *Dan Med J*. 2020;67(2)

Rahilly-Tierney C, Altincatal A, Agan A, et al. Linking Ambulance Trip and Emergency Department Surveillance Data on Opioid-Related Overdose, Massachusetts, 2017. *Public Health Rep*. Nov-Dec 2021;136(1_suppl):47s-53s. doi:10.1177/00333549211011626

Rajagopal S, Booth SJ, Brown TP, et al. Data quality and 30-day survival for out-of-hospital cardiac arrest in the UK out-of-hospital cardiac arrest registry: a data linkage study. *BMJ open*. 2017;7(11):e017784. doi:https://dx.doi.org/10.1136/bmjopen-2017-017784

Randell D. Documentation mnemonic and rubric substantially improved documentation. *Educator Update*. Winter2020 2020:13-16.

Redfield C, Tlimat A, Halpern Y, et al. Derivation and validation of a machine learning record linkage algorithm between emergency medical services and the emergency department. *J Am Med Inform Assoc*. Jan 1 2020;27(1):147-153. doi:10.1093/jamia/ocz176

Reisner AT, Chen L, Reifman J. The association between vital signs and major hemorrhagic injury is significantly improved after controlling for sources of measurement variability. *J Crit Care*. 2012;27(5):533.e1-533.e10. doi:10.1016/j.jcrc.2012.01.006

Richards CT, Mathew Li Z, Woodhouse A, et al. A pragmatic computer algorithm successfully matches de-identified regional quality improvement database records and emergency medical services records. *Stroke*. 2018;49(Supplement 1)American Heart Association/American Stroke Association 2018 International Stroke Conference and State-of-the-Science Stroke Nursing Symposium. Los Angeles, CA United States.

Robinson JB, Smith MP, Gross KR, et al. Battlefield Documentation of Tactical Combat Casualty Care in Afghanistan. *US Army Med Dept J*. 2016;(2-16):87-94.

Rykulski N, Berger D, Chen N-W, et al. Impact of missing data on measurement of cardiac arrest outcomes according to race. *Acad Emerg Med*. 2021;28(SUPPL 1):S265. Society for Academic Emergency Medicine Annual Meeting, SAEM 2021. Virtual. doi:http://dx.doi.org/10.1111/acem.14249

Savary D, Ricard Cc, Drouet A, et al. How exhaustive are out of hospital cardiac arrest registers? The example of the Northern French Alps Cardiac Arrest Registry. Elsevier B.V.; 2020. p. 57-58.

Saviluoto A, Björkman J, Olkinuora A, et al. The first seven years of nationally organized helicopter emergency medical services in Finland - the data from quality registry. *Scand J Trauma Resusc Emerg Med*. May 29 2020;28(1):46. doi:10.1186/s13049-020-00739-4

Schauer SG, April MD, Naylor JF, et al. A descriptive analysis of data from the Department of Defense Joint Trauma System Prehospital Trauma Registry. *US Army Med Dept J*. 2017;(3-17):92-97.

Scott EE, Krupa NL, Sorensen J, Jenkins PL. Electronic merger of large health care data sets: cautionary notes from a study of agricultural morbidity in New York State. *J Agromedicine*. 2013;18(4):334-9. doi:https://dx.doi.org/10.1080/1059924X.2013.826608

Seymour CW, Kahn JM, Angus DC, Martin-Gill C, Callaway CW, Yealy DM. Creating an infrastructure for comparative effectiveness research in emergency medical services. *Academic Emergency Medicine*. 2014;21(5):599-607. doi:http://dx.doi.org/10.1111/acem.12370

Silvestri S, Hunter C, Ralls G, Papa L. Capnography is a reliable method of determining endotracheal tube location in an out-of hospital cardiac arrest population confirmed by autopsy. *Acad Emerg Med*. 2012;19(SUPPL. 1):S259. 2012 Annual Meeting of the Society for Academic Emergency Medicine, SAEM 2012. Chicago, IL United States. doi:http://dx.doi.org/10.1111/j.1553-2712.2012.01332.x

Staff T, Søvik S. A retrospective quality assessment of pre-hospital emergency medical documentation in motor vehicle accidents in south-eastern Norway. *Scand J Trauma Resusc Emerg Med*. Mar 31 2011;19:20. doi:10.1186/1757-7241-19-20

Stephanian D, Brubacher J. Use of police and SAR records to identify cases and reduce survivorship bias in prehospital care research. *CJEM*. 2020;22(Supplement 1):S72. 2020 CAEP/ACMU. Ottawa, ON Canada. doi:http://dx.doi.org/10.1017/cem.2020.230

Stromsoe A, Svensson L, Axelsson AB, Goransson K, Todorova L, Herlitz J. Validity of reported data in the Swedish Cardiac Arrest Register in selected parts in Sweden. *Resuscitation*. 2013;84(7):952-6. doi:https://dx.doi.org/10.1016/j.resuscitation.2012.12.026

Sundermann ML, Salcido DD, Koller AC, Menegazzi JJ. Inaccuracy of patient care reports for identification of critical resuscitation events during out-of-hospital cardiac arrest. *Am J Emerg Med*. Jan 2015;33(1):95-9. doi:10.1016/j.ajem.2014.10.037

Swor R, Qu L, Putman K, et al. Challenges of Using Probabilistic Linkage Methodology to Characterize Post-Cardiac Arrest Care in Michigan. *Prehosp Emerg Care*. Mar-Apr 2018;22(2):208-213. doi:10.1080/10903127.2017.1362086

Tonsager K, Rehn M, Ringdal KG, et al. Collecting core data in physician-staffed pre-hospital helicopter emergency medical services using a consensus-based template: international multicentre feasibility study in Finland and Norway. *BMC Health Serv Res*. 2019;19(1):151. doi:https://dx.doi.org/10.1186/s12913-019-3976-6

Tonsager K, Kruger AJ, Ringdal KG, Rehn M. Data quality of Glasgow Coma Scale and Systolic Blood Pressure in scientific studies involving physician-staffed emergency medical services: Systematic review. *Acta anaesthesiologica Scandinavica*. 2020;64(7):888-909. doi:https://dx.doi.org/10.1111/aas.13596

Tainter F, Fitzpatrick C, Gazillo J, Riessman R, Knodler M, Jr. Using a novel data linkage approach to investigate potential reductions in motor vehicle crash severity - An evaluation of strategic highway safety plan emphasis areas. *J Safety Res*. 2020;74:9-15. doi:https://dx.doi.org/10.1016/j.jsr.2020.04.012

Therien SP, Nesbitt ME, Duran-Stanton AM, Gerhardt RT. Prehospital medical documentation in the Joint Theater Trauma Registry: a retrospective study. *J Trauma*. 2011;71(1 Suppl):S103-8. doi:https://dx.doi.org/10.1097/TA.0b013e3182218fd7

Timoteo MdSTBA, Dantas RAN, Costa ICS, et al. Implementation of improvement cycle in health records of mobile emergency prehospital care. *Revista brasileira de enfermagem*. 2020;73(4):e20190049. doi:https://dx.doi.org/10.1590/0034-7167-2019-0049

Tlimat A, Redfield C, Ullman EA, Nathanson LA, Horng S. Derivation and validation of a record linkage algorithm between EMS and the emergency department using machine learning. *Acad Emerg Med*. 2016;23(SUPPL. 1):S86-S87. 2016 Annual Meeting of the Society for Academic Emergency Medicine, SAEM 2016. New Orleans, LA United States. doi:http://dx.doi.org/10.1111/acem.12974

Tsur AM, Nadler R, Lipsky AM, et al. The Israel Defense Forces Trauma Registry: 22 years of point-of-injury data. *J Trauma Acute Care Surg*. 2020;89(2S Suppl 2):S32-S38. doi:https://dx.doi.org/10.1097/TA.0000000000002776

Wilharm A, Kulla M, Baacke M, et al. [Prehospital capnometry as quality indicator for trauma patients - Initial analysis from the TraumaRegister DGU.] *Anasthesiologie und Intensivmedizin*. 2019;60(9):419-432. Prahospitale Kapnometrie als Qualitatsindikator der Schwerverletztenversorgung Eine erste Auswertung aus dem TraumaRegister DGU. doi:http://dx.doi.org/10.19224/ai2019.419

Winter S, Jootun R. Audit of morphine administration by east midlands ambulance service (EMAS). *BMJ Open*. 2017;7(Supplement 3):A3-A4. 2nd European Emergency Medical Services Congress, EMS 2017. Copenhagen Denmark. doi:http://dx.doi.org/10.1136/bmjopen-2017-EMSabstracts.9
